# The Mother in the Factory

**Published:** 1898-03-26

**Authors:** 


### Modern Sociology.

### THE MOTHER IN THE FACTORY.—I.

In a very interesting paper, read before the Statistical Society on the 15th inst., Miss Clara Collet analyses the tables relating to the employment of women given in the census results, and draws some valuable inferences from them. Among other points, she deals at length on the effect on the health of children that results from their mothers being employed in some employment outside the home. Miss Collet admits that the whole question of the employment of married women cannot be settled by statistics; and it is obvious that a mother's moral influence, exercised in making the home clean, cheerful, and pleasant, must always be taken into account in any full discussion of the subject, as also the effect that women's work has on men, both as tending to lower wages and as developing lazy habits in those who are supported, wholly or partially, by their womenkind. But Miss Collet deals at length with the important question of the employment of mothers on the health of their infant children. In taking as a basis for her inferences the deaths of children under one year we think Miss Collet errs, as we can hardly put the limit of the time during which a child requires the constant attention of a mother so low; and, on the other hand, we should have been glad to have had the deaths of children under one month deducted from the totals, as for that first month of the child's life the Factory Acts forbid the employment of the mother. But it is doubtful if these facts, though not without value in themselves, would seriously affect Miss Collet's conclusions.

The popular opinion is that the infant mortality is highest in those districts where women are most largely employed outside the home; but it is worth while to make sure that the two matters are as intimately connected as is generally supposed. In a discussion on the subject before the Statistical Society, Mr. Noel Humphreys expressed the conviction that the existing statistics did not prove this contention. "Infant mortality is high in Lancashire and the West Riding of Yorkshire, where a large number of women are employed in factories," he said, "but it is higher in Durham and South Wales—mining districts—where women do not share to any extent in outside work. The increasing number of premature births had been attributed to the increasing employment of women, but, as Mr. Humphreys again pointed out, the death-rate from premature birth was higher in Norfolk and Suffolk, where only 20 per cent. of the women were engaged in some occupation, than in the districts previously mentioned, where the percentage of women workers ranged from 37 to 43. These statements Miss Collet's figures tend to confirm. She shows, indeed, that the infant death-rate is high in those towns where women are much employed in factory work. Thus, in Blackburn, where over 60 per cent. of the women are wage-earners, the deaths of infants during the period 1889—1893 amounted to 207 per 1,000 births; and in a series of towns the inverse progress is almost unbroken, the fewer the women employed in wage-earning work the greater the number of children who survive infancy, till in the case of Sunderland we find less than 25 per cent. of the women at work, and the infant death-rate fallen to 175 per 1,000. "There seems," says Miss Collet, "to be a kind of correspondence between employment of women and girls and infant mortality. But is this a relation of cause and effect? When it is here remembered that no account is here taken of kind of employment or conditions of work, of whether the majority employed are married or single, well-paid or ill-paid, we may well hesitate to come to such a conclusion on such slight evidence."

To arrive at a definite conclusion on the subject, we ought to deduct from the above totals unmarried women who are at work, while we admit that many female ailments, contracted through too severe or unsuitable work during youth, do not show their effects till after marriage. But the census returns do not differentiate between married and unmarried workers. Domestic servants, whose work is not in itself unhealthy, will also be included in these totals, and a separate analysis must be made of the various kinds of women's work before a reliable estimate can be made. It would be convenient if the distinction drawn by Professor Walker in his book on "Wages" were adopted in discussions of this point, and domestic servants were placed in the salaried class, by whose services their employer is not enriched, as distinct from the wage-earning class, from whose labours he gains a profit. All discussions of the employment of women are, in intention, confined to the latter class, but the statistics quoted usually include the former. But Miss Collet draws some original and suggestive inferences from the existence of a servant class. It appears that in those towns where the proportion of the female population to be found in domestic service is largest infant mortality is smallest. Thus, in Brighton, Birkenhead, and Bristol, where 13 per cent. of the female population over 10 years of age is so employed, the proportion of infant deaths is 153 per 1,000; while in such towns as Preston, Blackburn, and Bradford, where servants are in the proportion of only 7 per cent. of the females over 10, the deaths of children rise to 193 per 1,000. The proportion of servants to the population of a town is a fair clue to the average circumstances of the inhabitants. To keep a servant implies a certain superfluity of means beyond that required for the maintenance of one's own family. Those who keep servants do not live in overcrowded nor, as a rule, insanitary localities; their children are well fed and well cared for. Occasional instances of servants being kept by those who cannot afford to do so, through vanity, are so few, proportionately, that the existence of them cannot be held to affect the rule. "There is," as Miss Collet says, "a connection between small means and infant mortality, and between small means and the employment of women; but the latter relationship is not so close as the former, owing mainly to the migration of women to the wealthier districts as domestic servants."

In order to ascertain the number of married women who are employed in wage-earning work, Miss Collet has had recourse to the method employed in the labour department, viz., comparing the excess of occupied women over unmarried women between 25 and 45 years of age. This excess gives the minimum number of married women employed at that age-period, and from it the minimum percentage of such women who are also wage-earners can be ascertained. The statistics thus obtained show certainly an almost regularly descending series of infant deaths corresponding to the decrease in the employment of married women and widows.

